# Rifabutin disk diffusion on chelated Mueller-Hinton agar: a robust methodological framework for accurate *Acinetobacter baumannii* susceptibility testing

**DOI:** 10.1128/jcm.00598-26

**Published:** 2026-09-30

**Authors:** Michelle Zorawik, Jordyn VanOrman, Luke Bender, Lauren Tanner, Rosemary She, Brad Spellberg, Brian Luna

**Affiliations:** 1 Department of Immunology and Immune Therapeutics, Keck School of Medicine at USC 5116 , Los Angeles, California, USA; 2 Department of Pathology, City of Hope 378541, Duarte, California, USA; 3 Los Angeles General Medical Center 23336 , Los Angeles, California, USA; Mayo Clinic, Rochester, Minnesota, USA

**Keywords:** carbapenem-resistant *A. baumannii *(CRAB), rifabutin, Clinical and Laboratory Standards Institute (CLSI), disk diffusion, antimicrobial susceptibility testing

## Abstract

Carbapenem-resistant *Acinetobacter baumannii* (CRAB) is an extensively drug-resistant pathogen requiring new therapeutic strategies. Rifabutin (RBT), a U.S. Food and Drug Administration (FDA)-approved rifamycin, has demonstrated potent activity against CRAB; however, its activity is systematically underestimated by conventional antimicrobial susceptibility testing (AST) performed in conventional iron-rich Mueller-Hinton media, necessitating methods that accurately predict *in vivo* efficacy to guide its clinical use. Here, we describe the development of a disk diffusion method for RBT susceptibility testing against *A. baumannii*. Following Clinical and Laboratory Standards Institute (CLSI) M23 “Development of *In Vitro* Susceptibility Test Methods, Breakpoints, and Quality Control Parameters” guidance, multiple disk potencies were evaluated across standard and chelated Mueller-Hinton agar (MHA), including EDTA-supplemented media, and zone-of-inhibition (ZOI) diameters were correlated with MICs determined by agar dilution. A 20-µg RBT disk on chelated MHA, or equivalently on MHA supplemented with 100 mg/L EDTA, provided clear and reproducible discrimination of tentative wild-type and non-wild-type phenotypes, and preliminary quality control testing supported *A. baumannii* NCTC 13304 as a candidate quality control strain. While this investigational method has not been reviewed or approved by the CLSI, it was developed in concordance with the M23 guidance and addresses the key limitations of conventional AST, providing a robust framework for interlaboratory validation and future breakpoint determination.

**IMPORTANCE:**

Antimicrobial susceptibility testing is a critical tool for guiding clinical treatment decisions. Rifabutin exhibits potent activity against carbapenem-resistant *Acinetobacter baumannii* (CRAB) under the iron-restricted conditions encountered *in vivo*; however, its activity is systematically underestimated by standard testing methods in iron-rich Mueller-Hinton media. Here, we describe a robust, simple, and reproducible disk diffusion method using a 20-µg rifabutin disk on chelated Mueller-Hinton agar, which enables accurate assessment of rifabutin activity against *A. baumannii*. Our method provides a practical foundation for standardized rifabutin susceptibility testing, future breakpoint development, and broader evaluation of rifabutin against CRAB to ultimately support its implementation in clinical microbiology laboratories.

## INTRODUCTION

Carbapenem-resistant *Acinetobacter baumannii* (CRAB) is an extensively drug-resistant pathogen responsible for severe nosocomial infections, with mortality rates exceeding 50% in critically ill patients (1, 2). Designated by the World Health Organization as a top-priority threat, CRAB urgently requires new therapeutic strategies (3).

Rifabutin (RBT) is a U.S. Food and Drug Administration (FDA)-approved rifamycin originally developed for mycobacterial infections that has demonstrated remarkable potency against CRAB, far exceeding that of rifampicin (4–7). This enhanced activity is mediated by the outer-membrane receptor FhuE, a TonB-dependent siderophore transport system upregulated in iron-limited environments that facilitates active intracellular accumulation of RBT, thereby enabling potent inhibition of RNA polymerase (4, 5, 7). Such iron-limited environments are encountered *in vivo* but not in the nutrient-rich Mueller-Hinton media used in conventional antimicrobial susceptibility testing (AST) (8, 9). As a result, RBT activity is systematically underestimated by conventional testing conditions (4).

AST serves as the foundation for clinical antibiotic selection, with standardized methods and interpretive susceptibility breakpoints defined by the Clinical and Laboratory Standards Institute (CLSI) and the European Committee on Antimicrobial Susceptibility Testing (EUCAST) (10–12). Efforts to standardize RBT susceptibility testing against *A. baumannii* have centered on MIC testing (13), specifically agar dilution performed on Mueller-Hinton agar (MHA) supplemented with the iron chelator pyridoxal isonicotinoyl hydrazone (PIH), which has demonstrated robustness in predicting *in vivo* RBT activity and is anticipated to become the reference method (5, 14). However, preparation of agar plates containing multiple antibiotic concentrations is labor-intensive and impractical for routine use in clinical microbiology laboratories (15, 16). Consequently, a practical method capable of accurately predicting RBT activity while remaining feasible for routine use is needed.

Here, we describe the development of a disk diffusion method for RBT against *A. baumannii*, with the goal of providing a simple, practical, and reproducible approach for routine susceptibility testing (17). Following CLSI M23 “Development of *In Vitro* Susceptibility Test Methods, Breakpoints, and Quality Control Parameters” guidance for disk diffusion method development and quality control (QC) establishment (18, 19), multiple disk potencies were evaluated across different medium types and manufacturing lots, including standard and chelated Mueller-Hinton agar (CHE-MHA). Correlation of zone of inhibition (ZOI) measurements with agar dilution MICs identified the 20-µg RBT disk on CHE-MHA as reliable for differentiating tentative wild-type (WT) and non-wild-type (NWT) susceptibility phenotypes. EDTA-mediated chelation provided a practical alternative to resin-chelated media, and preliminary QC testing supported *A. baumannii* NCTC 13304 as a candidate QC strain. Although this investigational method has not been reviewed or approved by the CLSI, the present study was conducted in concordance with the M23 manual and establishes a robust foundation for expanded isolate testing, interlaboratory validation, and future breakpoint development to support its implementation in clinical microbiology laboratories.

## MATERIALS AND METHODS

### Bacterial isolates and genomic analysis

The complete panel of *A. baumannii* clinical isolates tested in this study is listed in Table S1. Isolates were obtained from the CDC and FDA Antimicrobial Resistance Isolate Bank (AR isolates), BEI Resources (HUMC1), and Dr. Robert Bonomo (UH516, Case Western Reserve University).

Whole-genome sequencing data were available for all isolates, except UH516. Existing NCBI genome assemblies were used when available, and assemblies for the remaining isolates were generated from publicly available Sequence Read Archive reads using SKESA v2.5.1.

Genetic determinants associated with reduced rifabutin activity and resistance were evaluated using NCBI BLAST+ v2.16.0+. *fhuE* sequences were extracted and compared with the characterized HUMC1 and LAC-4 *fhuE* alleles (4, 5), while *rpoB* sequences were compared with the HUMC1 *rpoB* sequence. The presence of the rifamycin ADP-ribosyltransferase gene *arr-2* was assessed using the *P. aeruginosa* reference sequence (GenBank accession no. AF078527.1).

### Antibiotic preparation

Antibiotic stock solutions were prepared on the day of each experiment by dissolving rifabutin powder (98% purity; Thermo Scientific, Cat. No. 461210050, Lot No. A0451198; and Cat. No. 461210010, Lot No. A0451198) in dimethyl sulfoxide (DMSO) at a concentration of 10 mg/mL. Working solutions were generated prior to use by diluting the rifabutin stock in phosphate-buffered saline (PBS).

### MIC testing by agar dilution

MICs for rifabutin were determined by agar dilution in accordance with CLSI M07 guidelines (13). A total of 30 *A. baumannii* clinical isolates were tested in triplicate across a concentration range of 0.001–32 mg/L, with *A. baumannii* NCTC 13304 tested in parallel to verify agar dilution MIC performance.

Testing was performed using Mueller-Hinton agar (MHA; prepared from Mueller Hinton broth powder [Criterion, Cat. No. C7521; Lot No. 637817] and bacteriological agar [Difco, Cat. No. B214530; Lot No. 4241155]) supplemented with 0.1 mM pyridoxal isonicotinoyl hydrazone (PIH; Abcam, Cat. No. AB145871; Lot No. APN14227-1-10). PIH was dissolved in DMSO and added to molten (~50°C) MHA to achieve the final concentration prior to antibiotic supplementation. Rifabutin working solutions were prepared at 100× the highest test concentration and serially diluted twofold to generate the range of concentrations used for testing. One hundred microliters of each working solution was added to 10-mL aliquots of molten PIH-supplemented MHA and poured into sterile Petri dishes to yield one plate per concentration, along with a drug-free plate serving as the growth control.

Bacterial inocula were prepared by suspending overnight colonies in phosphate-buffered saline to a turbidity equivalent to a 0.5 McFarland standard, followed by a 1:10 dilution in PBS. Two microliters of each suspension was manually applied to the surface of each agar plate, delivering approximately 10⁴ CFU per spot, starting with the drug-free control and proceeding in the order of increasing rifabutin concentration.

All assays were incubated at 35°C ± 2°C for 18–24 h under ambient conditions, and MICs were recorded as the lowest rifabutin concentration that visibly inhibited bacterial growth, disregarding the appearance of single colonies or very faint, indistinct hazes.

### Disk diffusion optimization

Disk diffusion assays were performed in accordance with CLSI M02 guidelines (20). Briefly, isolated overnight *A. baumannii* colonies were suspended in PBS to achieve turbidity equivalent to a 0.5 McFarland standard and uniformly inoculated onto the surface of standard MHA or CHE-MHA plates using a sterile cotton swab. Rifabutin disks were prepared in-house and applied to the agar surface, followed by incubation at 35°C ± 2°C in ambient air for 18–24 h. ZOIs were measured to the nearest millimeter at the inner margin of complete growth inhibition using a caliper (20).

Method optimization followed a two-phase approach, consisting of disk potency screening and MIC–ZOI correlation analysis, in accordance with CLSI M23S guidelines for the development of disk diffusion susceptibility testing methods (18, 19). To verify the performance of the prepared media and in-house rifabutin disks, quality control procedures were conducted, as described in the supplemental methods.

### Disk potency screening

A panel of 10 candidate RBT disk potencies (0.1, 0.2, 0.5, 1, 2, 5, 10, 20, 50, and 100 µg) was screened to narrow down concentrations suitable for susceptibility testing. Disks were prepared using a manual protocol consistent with CLSI M23S guidelines (19) by applying 20 µL of each 50× working solution, prepared from a single RBT powder source (98% purity; Thermo Scientific, Cat. No. 461210050; Lot No. A0451198), to the center of sterile 6-mm blank paper disks (HardyDisk Blank Disk, 0.25-inch diameter; Hardy Diagnostics, Cat. No. Z7121, Lot No. 515045). All disks were air-dried under sterile conditions and used immediately in disk diffusion assays.

Screening was performed using eight *A. baumannii* isolates, consisting of four tentative WT isolates (HUMC1, AR0273, AR0274, and AR0275) and four tentative NWT isolates, including two with rifabutin MICs of 4 mg/L (AR0284 and AR0306) and two with MICs of ≥32 mg/L (AR0299 and UH516). Assays were conducted on standard MHA (prepared from Mueller Hinton II broth powder, cation-adjusted [BD BBL, Cat. No. B12322; Lot No. 2343309] and bacteriological agar [Difco, Cat. No. B214530; Lot No. 4241155]) and on CHE-MHA (prepared from cation-depleted Mueller Hinton broth [Liofilchem, Cat. No. 400330; Lot No. 032524097] and bacteriological agar [Difco, Cat. No. B214530; Lot No. 4241155]).

ZOIs were measured as the inner diameter of complete growth inhibition and recorded to the nearest millimeter. Based on these measurements, four disk potencies were selected for extended MIC-ZOI correlation testing according to CLSI guideline criteria, including the following: (i) a general discriminatory power of 2–3 mm per log_₂_ MIC change among NWT isolates, (ii) inhibition zone diameters between 15 and 35 mm for WT isolates, (iii) optimal separation between WT and NWT isolates, and (iv) separation among NWT isolates with differing MICs.

### MIC-ZOI correlations

Four rifabutin disk potencies (5, 10, 20, and 50 µg) were selected for further evaluation using an expanded panel of 30 *A. baumannii* isolates to assess the correlation with MIC values and confirm ZOI consistency. Disks were prepared as described above, using two rifabutin powder sources in parallel (Thermo Scientific, 98% purity; Cat. No. 461210050; Lot No. A0451198; and Cat. No. 461210010; Lot No. A0451198) to address the absence of a commercially available rifabutin disk formulation.

Disk diffusion testing was performed using MHA prepared from four lots of Mueller-Hinton broth powder obtained from two manufacturers (BD BBL Mueller Hinton II, cation-adjusted, Cat. No. B12322; Lot Nos. 2343309 and 4102223; and Criterion Mueller Hinton broth, Cat. No. C7521; Lot Nos. 632186 and 637817) and bacteriological agar (Difco, Cat. No. B214530, Lot No. 4241155). Testing was also conducted using CHE-MHA prepared from two lots of cation-depleted Mueller Hinton broth (Liofilchem, Cat. No. 400330; Lot Nos. 032524097 and 041824033) and the same bacteriological agar. All lots of the same medium type were tested in parallel using the same 0.5 McFarland suspension for each corresponding isolate.

Zones of inhibition were measured to the nearest millimeter as the diameter of complete growth inhibition, and MIC-ZOI correlations were visualized using scatter plots and histograms. Statistical analyses were performed to evaluate differences in ZOI and MIC values by rifabutin product, medium type, and manufacturer using the Mann-Whitney *U* test. *P*-values ≤ 0.05 were considered statistically significant.

### Evaluation of EDTA-mediated chelation and growth kinetics

Rifabutin disk diffusion testing was repeated on MHA (prepared from Mueller Hinton II broth powder, cation-adjusted [BD BBL, Cat. No. B12322, Lot No. 2343309] and bacteriological agar [Difco, Cat. No. B214530, Lot No. 4241155]) supplemented with 100 mg/L ethylenediaminetetraacetic acid (EDTA, 0.5 M, Corning, Cat. No. 46-034-CI) to evaluate its suitability as a cost-effective alternative for CHE-MHA preparation. The same 30 *A. baumannii* isolates were tested in triplicate using 5-, 10-, 20-, and 50-µg rifabutin disks.

To assess whether EDTA supplementation affected bacterial viability or growth dynamics, colony growth was quantified using the ScanLag methodology as previously described (21). Briefly, *A. baumannii* AR_0298 was cultured overnight and diluted in PBS to an inoculum of approximately 1 × 10³ CFU/mL. In triplicate, 0.1 mL of the suspension was uniformly spread onto MHA or MHA supplemented with 100 mg/L EDTA, with a sterile piece of black felt cloth placed between the plate and lid to enhance colony contrast and absorb condensation during imaging.

Plates were incubated at 35°C ± 2°C under ambient atmospheric conditions for 24 h, and images were automatically captured every 15 min beginning at time zero using a flatbed scanner controlled by the ScanLag Manager software. Growth rates were quantified by measuring the time of colony expansion from 20 to 80 pixels for all replicates under each condition and visualized as histograms to compare growth kinetics between EDTA-supplemented and control media.

### Quality control establishment

QC verification was conducted in accordance with CLSI M23 guidelines for preliminary QC testing (Tier 1 studies) (21) to assess the suitability of *A. baumannii* NCTC 13304, an established CLSI QC strain for β-lactam combination agents (10) and the proposed rifabutin agar dilution QC strain for rifabutin disk diffusion testing.

Rifabutin MICs were confirmed using agar dilution on MHA supplemented with 0.1 mM PIH, as described previously. Disk diffusion QC testing of *A. baumannii* NCTC 13304 was performed using 20-µg rifabutin disks on CHE-MHA (prepared from cation-depleted Mueller Hinton broth [Liofilchem, Cat. No. 400330, Lot No. 032524097]) and bacteriological agar [Difco, Cat. No. B214530, Lot No. 4241155]) and on MHA (prepared from Mueller Hinton II broth powder, cation-adjusted [BD BBL, Cat. No. B12322, Lot No. 2343309] and bacteriological agar [Difco, Cat. No. B214530, Lot No. 4241155]) chelated with 100 mg/L EDTA, in parallel. A total of 20 independent replicates were conducted over 4 consecutive days (five replicates per day), each using a freshly prepared 0.5 McFarland inoculum to ensure independent testing. Zone diameters were measured to the nearest millimeter, and the mean, median, standard deviation, and 95% confidence interval around the median were calculated using the CLSI RangeFinder Disk tool to define a preliminary QC range in accordance with CLSI recommendations (22).

## RESULTS

### Rifabutin MIC distribution

Disk diffusion method development is informed by the distribution of MIC values obtained using standardized reference methods (18, 19). To inform rifabutin disk diffusion development and ensure an appropriate representation of susceptibility phenotypes, MICs were determined for 30 *A. baumannii* clinical isolates by agar dilution on PIH iron-chelated Mueller-Hinton agar.

**Fig 1:**
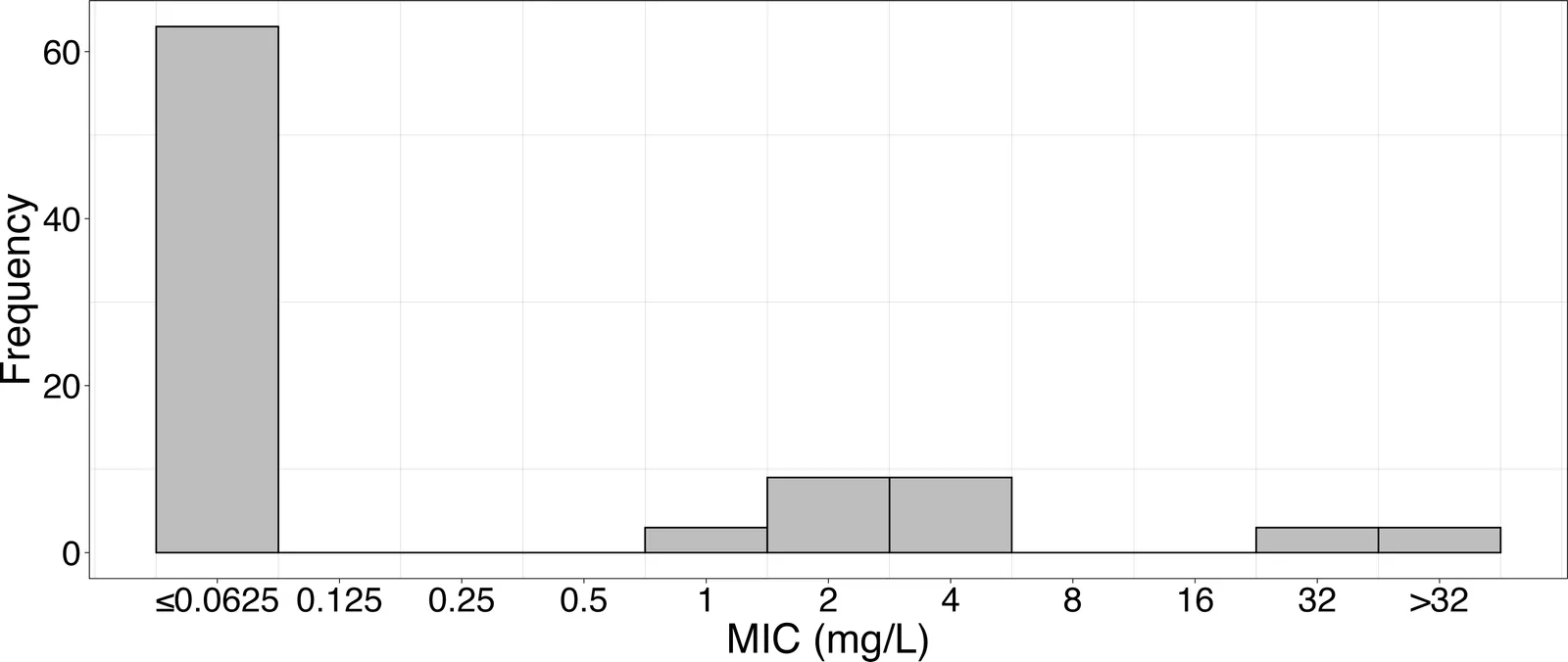
Rifabutin MIC distribution for *A. baumannii*. MICs for 30 *A. baumannii* clinical isolates were determined by agar dilution on Mueller-Hinton agar supplemented with 0.1 mM PIH. Testing was performed in triplicate across a concentration range of 0.001–32 mg/L, with values visualized from ≤ 0.0625 to >32 mg/L. Histograms display the frequency of isolates at each MIC value and serve as reference measurements for subsequent ZOI-MIC correlation analyses.

Testing yielded a trimodal MIC distribution consistent with previously described rifabutin susceptibility phenotypes (4, 5). Twenty-one of 30 isolates (70%) exhibited low MICs ≤ 0.0625 mg/L consistent with tentative WT profiles, supporting use of this value as a tentative WT cutoff (Fig. 1). The remaining nine isolates (30%) were classified as tentative NWT, comprising seven isolates with MICs of 1–4 mg/L and two isolates with MICs ≥ 32 mg/L.

Collectively, the panel provided a suitable range of MIC phenotypes to support CLSI-guided disk diffusion method development. Genomic analysis offered additional support for these tentative classifications. Whole-genome sequencing data were available for 29 of the 30 isolates. All 21 tentative WT isolates yielded full-length *fhuE* sequences that aligned closely with the HUMC1 allele associated with active rifabutin uptake. In contrast, only partial *fhuE* sequences were recovered from all eight sequenced tentative NWT isolates, and most recovered regions aligned more closely with the LAC-4 *fhuE* allele, which encodes an FhuE variant associated with impaired rifabutin uptake. No consistent *rpoB* sequence pattern distinguished tentative WT from NWT isolates. Among the sequenced isolates, *arr-2* was detected only in AR0299, the only sequenced tentative NWT isolate with a rifabutin MIC > 32 mg/L.

### Rifabutin disk potency screening

Disk diffusion development requires selection of a disk potency that yields interpretable zone diameters and reliably separates isolates across susceptibility categories (19). To narrow down suitable rifabutin concentrations for *A. baumannii*, an initial screen of 10 disk potencies (0.1–100 µg) was performed on standard MHA and on chelated MHA (CHE-MHA) using four tentative WT and four NWT isolates to determine 2–4 potencies demonstrating the greatest discriminatory power.

As summarized in Table 1 for MHA and Table 2 for CHE-MHA, potencies ≤ 2 µg produced tentative WT inhibition zones below the 15–35 mm target range, while ZOI increases between successive potencies were greatest within the 2–50 µg interval. On MHA, potencies ≥ 10 µg consistently generated acceptable zone diameters (15–30 mm) for tentative WT isolates, with the 50-µg disk providing the widest ZOI separation (21 mm) between tentative WT and NWT isolates. On CHE-MHA, WT zones remained within range at 5–100 µg (18–30 mm), and the 10-µg disk maximally separated tentative WT and NWT isolates (18-mm difference). Optimal performance was therefore suggested for RBT disks of 50 µg on MHA and 10 µg on CHE-MHA, leading to the selection of four candidate potencies (5, 10, 20, and 50 µg) for extended MIC-ZOI correlation testing.

**TABLE 1:** Inhibition zone diameters (in mm) and differences by rifabutin disk content for *A. baumannii* on MHA

Isolate ID	MIC, mg/L^*a*^	Tentative WT/NWT status^*b*^	Rifabutin disk content (potency), µg^*c*^									
			0.1	0.2	0.5	1	2	5	10	20	50	100
HUMC1	≤0.0625	WT	6	6	6	6	10	18	22	24	28	30
AR0273	≤0.0625	WT	6	6	6	6	11	19	21	21	24	26
AR0274	≤0.0625	WT	6	6	6	6	7	14	15	17	22	25
AR0275	≤0.0625	WT	6	6	6	6	8	15	20	22	24	27
AR0284	4	NWT	6	6	6	6	6	9	12	14	17	19
AR0306	4	NWT	6	6	6	6	6	8	11	14	18	20
AR0299	>32	NWT	6	6	6	6	6	6	6	7	11	14
UH516	32	NWT	6	6	6	6	6	6	6	6	7	9
Difference between largest and smallest zone			0	0	0	0	5	13	16	18	**21**	21

^*a*^ Determined by agar dilution on MHA supplemented with 0.1 mM PIH.

^*b*^ Assigned using ≤0.0625 mg/L as the tentative WT MIC cutoff.

^*c*^ Disk contents (potencies) with both optimal zone sizes and the largest increase in zone sizes between two consecutive disk contents (potencies) for tentative WT isolates are highlighted in gray. The greatest difference (mm) between the largest and smallest zone within the optimal area is in bold.

**TABLE 2:** Inhibition zone diameters (in mm) and differences by rifabutin disk content for *A. baumannii* on CHE-MHA

Isolate ID	MIC, mg/L^*a*^	Tentative WT/NWT status^*b*^	Rifabutin disk content (potency), µg^*c*^									
			0.1	0.2	0.5	1	2	5	10	20	50	100
HUMC1	≤0.0625	WT	6	6	6	6	10	18	22	24	28	30
AR0273	≤0.0625	WT	6	6	6	6	12	19	21	21	25	27
AR0274	≤0.0625	WT	6	6	6	6	17	21	22	23	25	26
AR0275	≤0.0625	WT	6	6	6	6	15	20	24	24	25	29
AR0284	4	NWT	6	6	6	6	6	10	15	16	19	22
AR0306	4	NWT	6	6	6	6	6	10	14	16	20	21
AR0299	>32	NWT	6	6	6	6	6	6	6	8	11	13
UH516	32	NWT	6	6	6	6	6	6	6	7	11	13
Difference between largest and smallest zone			0	0	0	0	11	15	**18**	17	17	17

^*a*^ Determined by agar dilution on MHA supplemented with 0.1 mM PIH.

^*b*^ Assigned using ≤0.0625 mg/L as the tentative WT MIC cutoff.

^*c*^ Disk contents (potencies) with both optimal zone sizes and the largest increase in zone sizes between two consecutive disk contents (potencies) for tentative WT isolates are highlighted in gray. The greatest difference (mm) between the largest and smallest zone within the optimal area is in bold.

### MIC-ZOI correlations

Identification of the optimal rifabutin disk potency was performed by correlating ZOI measurements from four candidate disk concentrations (5, 10, 20, and 50 µg) with agar dilution MICs across the full panel of *A. baumannii* isolates (*n* = 30). Testing was conducted on standard MHA and CHE-MHA, using multiple medium manufacturer lots and two rifabutin powder sources in parallel to evaluate inter-manufacturer and inter-lot consistency.

Across both media, tentative WT isolates consistently produced inhibition zones with faint peripheral halos 3 to 5 mm that occasionally contained small colonies. These partial inhibition features were absent in tentative NWT isolates and were accounted for by measuring the inner margin of complete inhibition (Fig. S1).

**Fig 2:**
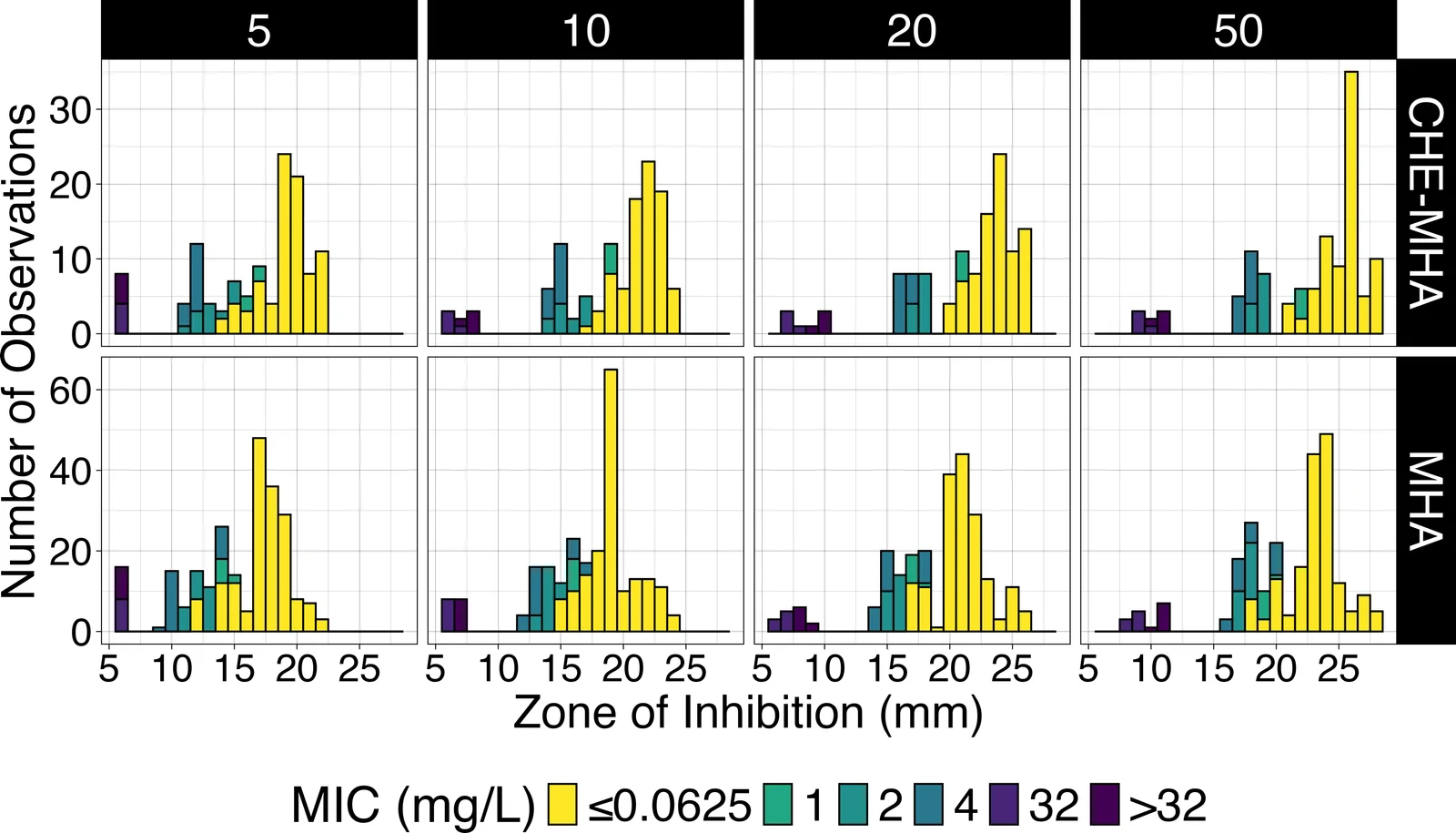
Rifabutin disk diffusion in *A. baumannii* requires CHE-MHA. Histograms show that the rifabutin zone of inhibition diameters (mm) correlated with agar dilution MICs determined on PIH-supplemented MHA. ZOIs were measured on chelated MHA (top) or standard MHA (bottom). Each *A. baumannii* isolate (*n* = 30) was tested with 5-, 10-, 20-, and 50-µg rifabutin disks prepared from two drug sources and applied to two lots of CHE-MHA, yielding four ZOI-MIC measurements per isolate; standard MHA testing included two lots each from two manufacturers, yielding eight measurements per isolate. Bar colors denote MIC categories, with yellow representing tentative WT isolates and darker colors representing tentative NWT isolates with progressively higher MICs. Clear separation of tentative WT and NWT populations was observed only on chelated MHA.

A clear separation between tentative WT and NWT isolates was observed only on CHE-MHA (Fig. 2). Tentative WT isolates (MIC ≤0.0625 mg/L) exhibited ZOIs ≥ 20 mm when tested with rifabutin disks containing ≥20 µg, visibly separating them from tentative NWT isolates. In contrast, none of the disk potencies tested on standard MHA differentiated tentative WT and NWT populations. On standard MHA, tentative WT isolates displayed broadened ZOI distributions that overlapped substantially with tentative NWT isolates in the 16–20 mm range, indicating that standard MHA lacks the iron-depleted conditions required for rifabutin activity in *A. baumannii*. No statistically significant differences in ZOI distributions were observed between rifabutin products or medium manufacturer lots (Mann-Whitney *U* test, *P* > 0.05) (Fig. S2 to S7).

**Fig 3:**
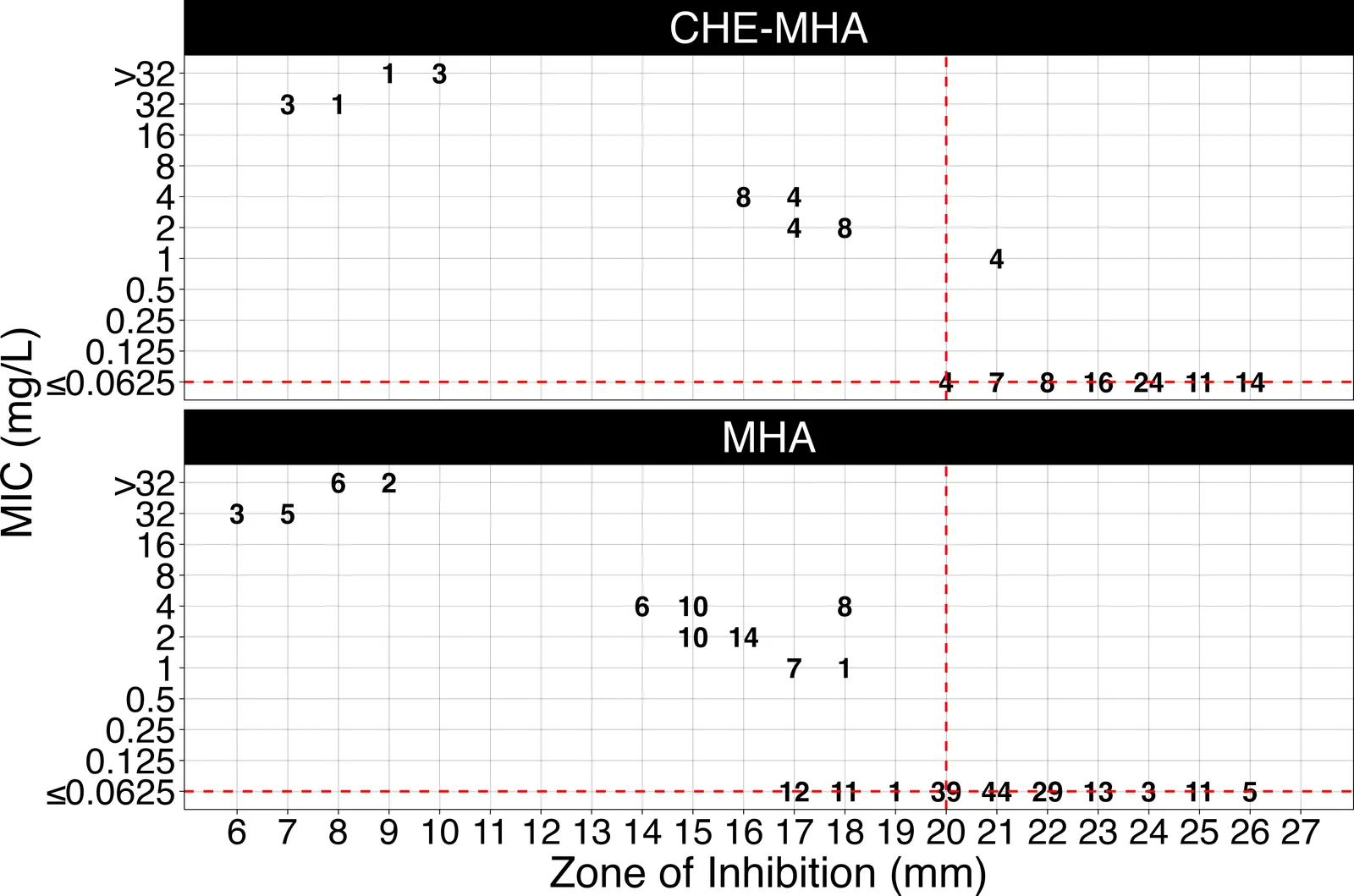
A 20-µg rifabutin disk on CHE-MHA optimally separates tentative WT and NWT phenotypes. Scatterplots show zone of inhibition diameters (mm) from rifabutin disk diffusion testing using a 20-µg disk on chelated MHA (top) or standard MHA (bottom), plotted against MIC values (mg/L) determined by agar dilution on PIH-supplemented MHA. Each label indicates the number of observations for a given ZOI-MIC combination, while red dashed reference lines denote the tentative WT cutoff (≤0.0625 mg/L) and corresponding ZOI threshold (≥20 mm). Rifabutin MICs correlate strongly with ZOIs generated on CHE-MHA, enabling clear ZOI-based discrimination of tentative WT and NWT phenotypes that was not achieved on standard MHA.

Among the four candidate disk potencies, the 20-µg rifabutin disk on CHE-MHA demonstrated optimal performance, showing strong MIC-ZOI correlations and clear phenotypic separation (Fig. 3). Tentative WT isolates (MIC ≤0.0625 mg/L) produced ZOIs of 20–26 mm, tentative NWT isolates with MICs of 1–4 mg/L exhibited zones of 16–18 mm, and tentative NWT isolates with MICs ≥ 32 mg/L showed minimal or no inhibition (6–10 mm). Although the 50-µg disk exhibited similar discriminatory power, it provided limited additional separation, increasing ZOIs by approximately 2 mm per isolate. Collectively, these findings demonstrate that the 20-µg rifabutin disk on CHE-MHA provides robust discrimination of tentative WT and NWT phenotypes represented in this study, supporting its use for subsequent QC range establishment and breakpoint determination.

### Evaluation of EDTA-chelated MHA

Accurate rifabutin susceptibility testing requires iron-depleted conditions to enable drug activity against *A. baumannii* (4, 5). The cation-depleted Mueller-Hinton broth used to prepare CHE-MHA in this study is commercially available and is chelated using a Chelex resin consistent with the CLSI-described method for producing iron-depleted cation-adjusted Mueller-Hinton broth for cefiderocol broth microdilution testing (23, 24). However, Chelex treatment is time- and labor-intensive, and reliance on specialized media may limit routine implementation in some clinical laboratories. EDTA, a broad-spectrum chelating agent with high affinity for divalent cations, including iron (25), offers a potential alternative for iron depletion through direct supplementation of standard media. To evaluate its suitability for RBT disk diffusion, MHA was supplemented with 100 mg/L EDTA and assessed for effects on bacterial growth and disk performance.

**Fig 4:**
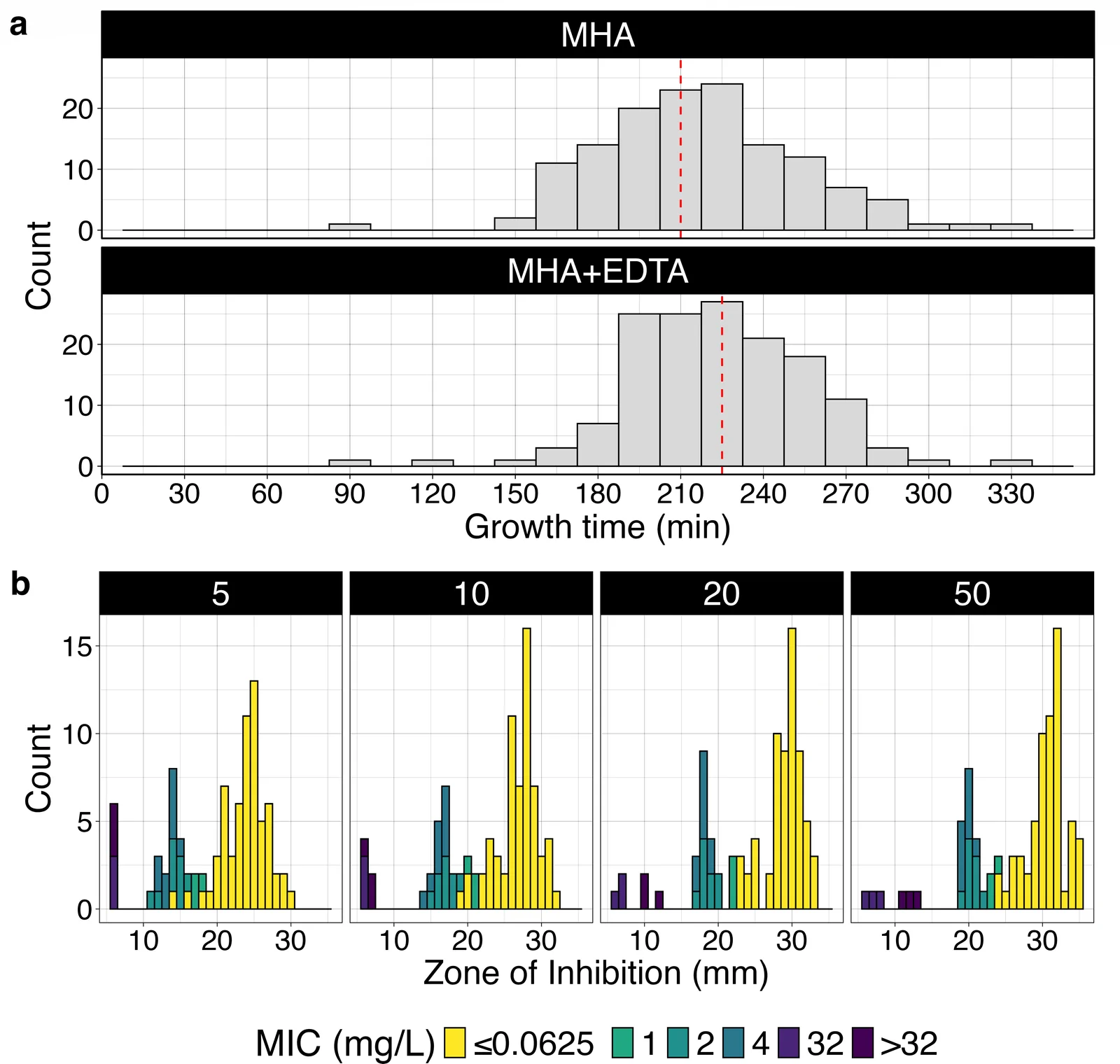
EDTA-chelated MHA offers a practical alternative to CHE-MHA for rifabutin disk diffusion. (**a**) ScanLag analysis of *A. baumannii* AR_0298 plated on standard MHA (top) or MHA supplemented with 100 mg/L EDTA (bottom). Growth time was defined as the interval required for individual colonies to expand from 20 to 80 pixels, measured by automated imaging every 15 min and converted to minutes. Median growth times are indicated by red dashed lines. No statistically significant difference was detected between growth on MHA vs MHA+EDTA (Mann–Whitney *U*, *P* = 0.0668). (**b**) Rifabutin zone of inhibition distributions on MHA supplemented with 100 mg/L EDTA. *A. baumannii* isolates (*n* = 30) were tested in triplicate using rifabutin disk potencies of 5, 10, 20, and 50 µg, and ZOI diameters were correlated with MIC values determined by agar dilution. Disk diffusion testing with a 20-µg rifabutin disk enabled clear ZOI-based discrimination of tentative WT and NWT phenotypes, consistent with results obtained using CHE-MHA.

ScanLag analysis of *A. baumannii* AR_0298 demonstrated comparable colony growth kinetics between MHA and MHA+EDTA (Fig. 4a). Based on 15-min imaging intervals, the median colony growth time from 20 to 80 pixels was 210 min on MHA (*n* = 136) and 225 min on MHA+EDTA (*n* = 145). The difference in growth rates was not statistically significant (Mann–Whitney *U*, *P* = 0.0668), confirming that EDTA supplementation at 100 mg/L is not inhibitory and supporting its further evaluation for rifabutin disk diffusion.

Disk diffusion testing on MHA+EDTA was performed using 5-, 10-, 20-, and 50-µg rifabutin disks against the same 30 *A. baumannii* isolates evaluated previously. ZOIs correlated well with agar dilution MICs and demonstrated performance comparable to that of CHE-MHA, with similar zone diameters observed across isolates (Fig. 4b). The 20-µg rifabutin disk provided optimal discrimination between susceptibility phenotypes, with tentative WT isolates (MIC ≤0.0625 mg/L) producing ZOIs ≥ 22 mm (observed range: 22–33 mm), consistent with the CLSI-recommended WT target range of 15–35 mm. Tentative NWT isolates with MICs of 1–4 mg/L exhibited ZOIs of 17–20 mm, whereas tentative NWT isolates with MICs ≥ 32 mg/L showed minimal or absent inhibition (≤12 mm).

These findings demonstrate that direct supplementation of MHA with 100 mg/L EDTA provides sufficient iron chelation to reproduce the discriminatory performance of CHE-MHA without significantly altering bacterial growth, supporting its use as a practical alternative for rifabutin disk diffusion testing.

### Rifabutin disk diffusion QC

**Fig 5:**
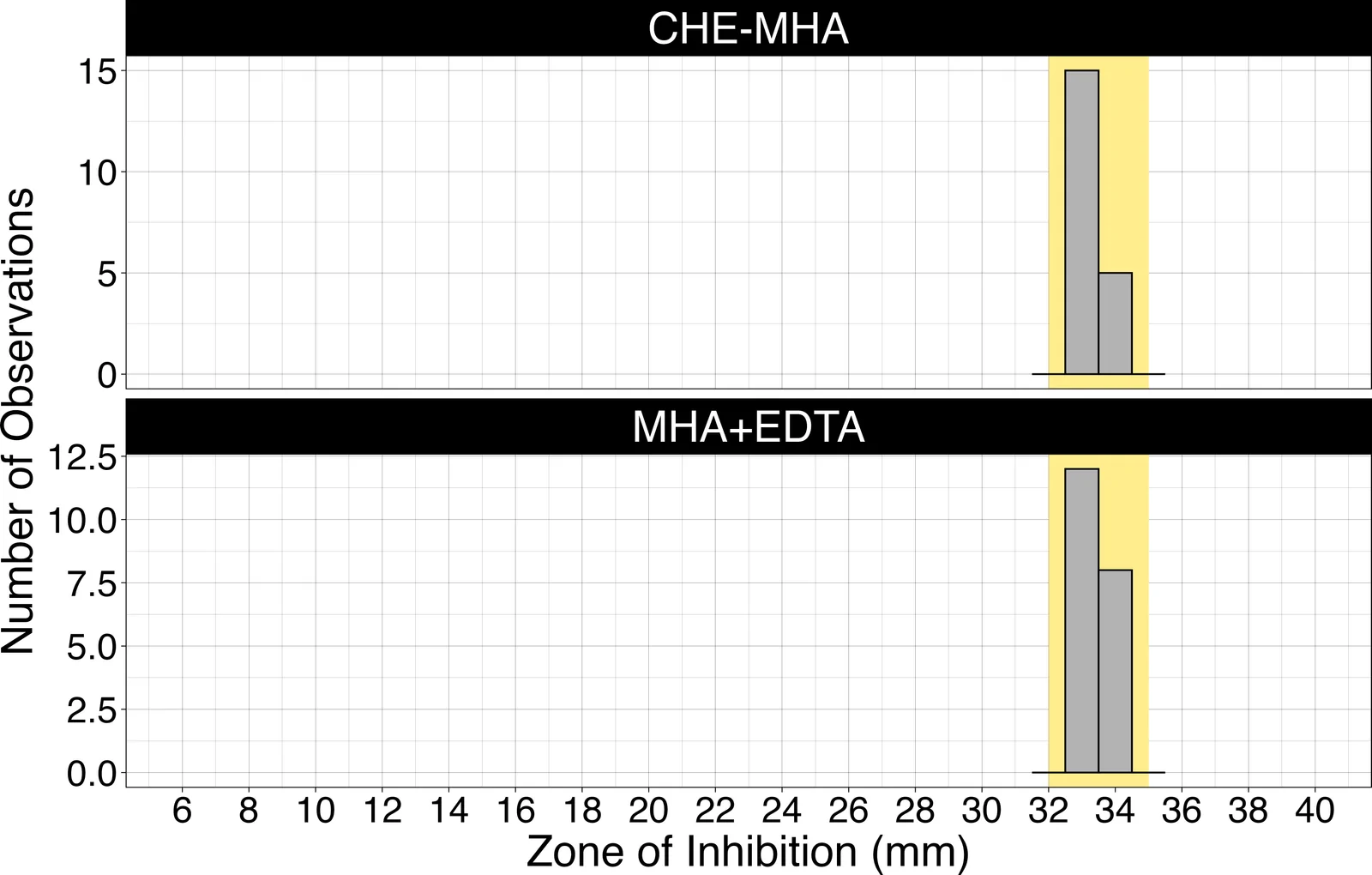
Preliminary quality control testing supports *A. baumannii* NCTC 13304 as a candidate QC strain for rifabutin disk diffusion. Zone of inhibition histograms for *A. baumannii* NCTC 13304 tested with a 20-µg rifabutin disk on CHE-MHA (top) and MHA supplemented with 100 mg/L EDTA (bottom). Disk diffusion QC testing was performed in accordance with CLSI M23, with 20 independent replicates tested in parallel on both media over 4 consecutive days. ZOI measurements from each medium were analyzed using the CLSI RangeFinder Disk tool. Both media yielded the same tentative QC range of 32–35 mm (highlighted in yellow), supporting MHA+EDTA as a practical alternative to CHE-MHA for rifabutin QC testing.

Preliminary quality control verification was conducted in accordance with CLSI M23 guidelines (18) to evaluate *A. baumannii* NCTC 13304 as a candidate rifabutin disk diffusion QC strain. Testing of 20 independent replicates using 20-µg RBT disks on CHE-MHA and on MHA+EDTA showed a narrow ZOI diameter range of 33–34 mm on CHE-MHA, with a mean of 33.3 mm, median and mode of 33 mm, and a standard deviation of 0.44 mm. The 95% confidence interval (32.4–34.1 mm) supported a tentative QC range of 32–35 mm (Fig. 5, top).

MHA+EDTA produced nearly identical results, with zone diameters of 33–34 mm, a mean of 33.4 mm, median and mode of 33 mm, and a standard deviation of 0.50 mm (Fig. 5, bottom). The 95% confidence interval (32.4–34.4 mm) likewise yielded a tentative QC range of 32–35 mm, demonstrating highly reproducible ZOIs across both media that meet CLSI criteria for QC range establishment and supporting *A. baumannii* NCTC 13304 as a candidate QC strain for rifabutin disk diffusion.

## DISCUSSION

Rifabutin, an FDA-approved rifamycin antibiotic with well-characterized pharmacologic properties, has gained increasing attention as a potential therapy for carbapenem-resistant *A. baumannii* infections (26). It exhibits potent intrinsic activity against CRAB, and RBT–polymyxin combinations have demonstrated a significant survival benefit with reduced emergence of resistance in murine infection models (27), whereas clinical studies of rifampicin-based combinations failed to demonstrate improved survival despite reductions in microbial burden (28). These findings have supported continued translational development of RBT, including formulation of an intravenous preparation (BV100) (7) and completion of a phase II clinical evaluation assessing its pharmacokinetics and safety in combination with polymyxin B in adult patients with CRAB-associated ventilator-associated pneumonia (NCT05685615). However, RBT activity remains systematically underestimated by conventional AST owing to its iron-dependent uptake via the outer membrane receptor FhuE (4, 5).

This study followed CLSI M23 guidelines (18, 19) to establish key parameters for RBT disk diffusion testing, providing a simple and robust methodological framework for accurate assessment of RBT activity in *A. baumannii*. While significant progress has been made toward establishing agar dilution on MHA supplemented with the iron chelator PIH as the reference MIC method for RBT susceptibility testing (5, 14), the approach is labor-intensive and not feasible for routine use in clinical settings (15, 16).

Method development used a two-phase screening strategy, with disk potency and medium selection informed by agar dilution MICs. MIC testing classified 70% of isolates as tentative WT and 30% as tentative NWT, meeting CLSI M23 method-development criteria for inclusion of 50%–80% WT isolates. Genomic findings provided support for these classifications and were consistent with previously described mechanisms affecting rifabutin activity (4, 5). The association of tentative WT phenotypes with full-length *fhuE* sequences closely aligned with the HUMC1 allele and tentative NWT phenotypes with incomplete *fhuE* sequence recovery, with most regions showing greater similarity to the LAC-4 allele, is consistent with FhuE-mediated uptake as a contributor to elevated MICs. The absence of a consistent *rpoB* sequence pattern and detection of *arr-2* in the high-MIC isolate AR0299 further suggests that multiple mechanisms may contribute to tentative NWT phenotypes.

Nevertheless, in the absence of established RBT breakpoints or sufficient MIC distribution data to define tentative epidemiological cutoff values (29), the tentative WT cutoff used in this study was informed by previously published MIC and *in vivo* efficacy data for *A. baumannii* isolates (4). While these data provide a meaningful reference point, the panel lacked isolates with MICs > 0.0625 and <1 mg/L, limiting evaluation immediately above the tentative WT cutoff. Although isolates within this interval appear to be relatively uncommon (5), their absence could influence selection of the optimal disk potency if the eventual WT distribution or epidemiological cutoff value extends above 0.0625 mg/L. Accordingly, testing across larger isolate panels that include MICs within this interval will be required to refine the WT distribution and validate the tentative cutoff.

Initial phase I experiments evaluated a broad range of RBT disk concentrations to identify candidate potencies capable of differentiating susceptibility phenotypes. Four disk potencies were subsequently selected for expanded evaluation in phase II testing across a larger isolate panel and multiple medium conditions. Phase II testing identified the 20-µg RBT disk on CHE-MHA as the optimal potency for disk diffusion testing. ZOI diameters on CHE-MHA correlated strongly with MICs and clearly distinguished tentative WT and NWT phenotypes across the MIC range represented in this study.

Notably, clear phenotypic separation was achieved only on CHE-MHA. Although agar intrinsically binds iron (30), RBT failed to differentiate tentative WT from tentative NWT isolates on standard MHA, suggesting that its iron content (~0.5 mg/L) (31) exceeds the threshold required for accurate assessment of RBT activity in *A. baumannii*. This contrasts with cefiderocol, another antibiotic whose activity depends on iron restriction and for which MHA is CLSI-approved for disk diffusion testing (23, 32). However, variability in ZOI diameters between manufacturers has been observed and attributed to differences in iron content between medium sources (33, 34). The consistent performance of RBT disks across reagent and medium lots in this study supports the robustness of the proposed method when iron limitation is appropriately controlled and underscores the importance of additional iron depletion to ensure accurate susceptibility testing for iron-dependent antibiotics.

Although the cation-depleted Mueller-Hinton broth used to prepare CHE-MHA is commercially available, reliance on specialized media may limit routine implementation in some clinical laboratories. Alternatively, cation-depleted broth can be prepared through in-house Chelex treatment; however, the process is time-consuming and labor-intensive (23, 24). MHA supplemented with 100 mg/L EDTA was, therefore, evaluated as a simpler and potentially more broadly accessible approach to media chelation. EDTA strongly binds and sequesters divalent cations in aqueous solutions, including iron; however, excessive concentrations can impair membrane integrity and disrupt bacterial growth (35, 36). Consistent with prior reports identifying ≤512 mg/L as sub-inhibitory for *A. baumannii* (37), ScanLag analysis showed that 100 mg/L EDTA did not significantly alter colony growth kinetics. Subsequent disk diffusion testing on MHA supplemented with EDTA reproduced the performance of CHE-MHA. The 20-µg RBT disk maintained clear separation between tentative WT and NWT isolates, with ZOI diameters increasing modestly (approximately 2–3 mm per isolate) while remaining within CLSI precision limits (±3 mm) (10, 20). Together, these findings demonstrate that direct EDTA supplementation of MHA provides sufficient iron chelation to enable RBT activity without significantly altering bacterial growth, offering a practical alternative to CHE-MHA. Verification across additional isolates and medium lots will be required to confirm these findings.

Preliminary QC testing supported *A. baumannii* NCTC 13304 as a candidate QC strain for RBT disk diffusion on CHE-MHA and MHA supplemented with EDTA, yielding reproducible ZOI diameters and a tentative QC range of 32–35 mm on both media. As this strain already serves as a CLSI QC organism for β-lactam combination agents (10), its future adoption for RBT disk diffusion could streamline implementation and facilitate standardization. Interlaboratory testing will be required to establish definitive QC ranges and confirm reproducibility across testing conditions.

Collectively, this study defines key parameters for RBT disk diffusion testing against *A. baumannii*. A 20-µg RBT disk applied to CHE-MHA, or equivalently to MHA supplemented with 100 mg/L EDTA, provided clear and reproducible discrimination of tentative WT and NWT phenotypes while maintaining a strong correlation with agar dilution-derived MICs obtained on PIH-chelated MHA. By accounting for FhuE-dependent iron uptake, this approach addresses limitations of conventional AST media that obscure RBT activity. Although the method has not been reviewed or approved by the CLSI, it establishes a practical framework for expanded isolate testing, interlaboratory validation, and future breakpoint development that may facilitate its eventual implementation in clinical laboratories to support reliable assessment of RBT activity against CRAB.

## Data Availability

Whole-genome sequencing data are publicly available through NCBI, and accession numbers are provided in Table S1.

## SUPPLEMENTAL MATERIAL

Supplemental materials jcm.00598-26-s0001.docx Supplemental methods, Table S1, and Fig. S1 to S7.

ASM does not own the copyrights to Supplemental Material that may be linked to, or accessed through, an article. The authors have granted ASM a non-exclusive, world-wide license to publish the Supplemental Material files. Please contact the corresponding author directly for reuse.
