# Supplementary material for: Rifabutin disk diffusion on chelated Mueller-Hinton agar: a robust methodological framework for accurate Acinetobacter baumannii susceptibility testing
Source: J Clin Microbiol. 2026 Sep 30;64(11):e00598-26. doi: 10.1128/jcm.00598-26 (PMC13753781; doi:10.1128/jcm.00598-26)
Supplement: Supplementary file 1 [file jcm.00598-26-s0001.docx]

# **Supplementary Methods**

## **Quality Control Validation of Media and Disk Preparations**

To confirm the reliability of all test media and the manual rifabutin disk preparation process, quality control testing was performed in accordance with CLSI M23S3 guidelines for evaluating Mueller-Hinton agar acceptability.^1^ Rifampin was used as a surrogate analyte due to its comparable antimicrobial properties and availability in both commercial and powdered formulations. Rifampin disks were prepared using the same manual protocol as described previously for rifabutin disk preparation, following CLSI M23S2, Appendix B.^2^

Manual rifampin disks (5 µg) were prepared from rifampin powder (Thermo Scientific, 95% purity, Cat. No. 455620050, Lot No. A0455669) and tested in parallel with commercial rifampin disks (BD BBL™ Sensi-Disc, 5 µg, Cat. No. 231541, Lot No. 3304649) using *Escherichia coli* ATCC 25922 and *Staphylococcus aureus* ATCC 29213. Zone of inhibition diameters obtained with manual and commercial disks were interpreted using CLSI M100^3^ performance ranges and compared to evaluate media workability and disk preparation reproducibility.

The pH, Ca²⁺/Mg²⁺ content, and Zn²⁺ availability of each agar source was further assessed using *Pseudomonas aeruginosa* ATCC 27853 by testing with commercial gentamicin (10-µg) and meropenem (10-µg) disks (BD BBL™ Sensi-Disc, Cat. Nos. 231227 and 231703; Lot Nos. 3303724 and 4033203).

# **Supplementary Data**

## **Table S1. Summary of clinical *A. baumannii* test isolates.**

| **No.** | **Isolate ID** | **Source** | **NCBI BioSample Accession Number** | **Carbapenem Susceptibility Phenotype** |
| --- | --- | --- | --- | --- |
| 1 | AR 0273 | AR Isolate Bank^†^ | SAMN04901663 | Resistant |
| 2 | AR 0274 | AR Isolate Bank | SAMN04901664 | Resistant |
| 3 | AR 0275 | AR Isolate Bank | SAMN04901665 | Resistant |
| 4 | AR 0276 | AR Isolate Bank | SAMN04901666 | Resistant |
| 5 | AR 0277 | AR Isolate Bank | SAMN04901667 | Resistant |
| 6 | AR 0279 | AR Isolate Bank | SAMN04901669 | Resistant |
| 7 | AR 0281 | AR Isolate Bank | SAMN04901671 | Resistant |
| 8 | AR 0282 | AR Isolate Bank | SAMN04901672 | Resistant |
| 9 | AR 0283 | AR Isolate Bank | SAMN04901673 | Resistant |
| 10 | AR 0284 | AR Isolate Bank | SAMN04901674 | Resistant |
| 11 | AR 0285 | AR Isolate Bank | SAMN04901675 | Resistant |
| 12 | AR 0286 | AR Isolate Bank | SAMN04901676 | Resistant |
| 13 | AR 0290 | AR Isolate Bank | SAMN04901680 | Resistant |
| 14 | AR 0291 | AR Isolate Bank | SAMN04901681 | Resistant |
| 15 | AR 0293 | AR Isolate Bank | SAMN04901683 | Resistant |
| 16 | AR 0295 | AR Isolate Bank | SAMN04901685 | Resistant |
| 17 | AR 0296 | AR Isolate Bank | SAMN04901686 | Resistant |
| 18 | AR 0297 | AR Isolate Bank | SAMN04901687 | Resistant |
| 19 | AR 0298 | AR Isolate Bank | SAMN04901688 | Resistant |
| 20 | AR 0299 | AR Isolate Bank | SAMN04901689 | Resistant |
| 21 | AR 0300 | AR Isolate Bank | SAMN04901690 | Susceptible |
| 22 | AR 0301 | AR Isolate Bank | SAMN04901691 | Resistant |
| 23 | AR 0302 | AR Isolate Bank | SAMN04901692 | Resistant |
| 24 | AR 0304 | AR Isolate Bank | SAMN04901694 | Resistant |
| 25 | AR 0306 | AR Isolate Bank | SAMN04901696 | Resistant |
| 26 | AR 0307 | AR Isolate Bank | SAMN04901697 | Resistant |
| 27 | AR 0309 | AR Isolate Bank | SAMN04901699 | Resistant |
| 28 | AR 0310 | AR Isolate Bank | SAMN04901700 | Resistant |
| 29 | HUMC1 | BEI Resources | SAMN45068614 | Resistant |
| 30 | UH516 | Case Western Reserve University | N/A^‡^ | Resistant |
| ^†^ AR Isolate Bank = CDC and FDA Antimicrobial Resistance Isolate Bank. | | | | |
| ^‡^ N/A = Not available. | | | | |

**Figure S1. Rifabutin zone of inhibition phenotypes and measurement on CHE-MHA.**

Representative disk diffusion images of 5-, 10-, 20-, and 50-µg rifabutin disks tested against *A. baumannii* isolates classified as **(a)** tentative WT (MIC ≤0.0625 mg/L), **(b)** tentative NWT with MICs of 1-4 mg/L, and **(c)** tentative NWT with MICs ≥32 mg/L. Red horizontal lines across zones produced by the 20-µg disks indicate the ZOI diameter measured at the inner margin of complete growth inhibition. Peripheral halos of partial growth, which were consistently observed around zones produced by tentative WT isolates, were excluded from ZOI measurements.

## **Figure S2. ZOI–MIC correlations by rifabutin product source on CHE-MHA.**

Histograms show ZOI diameters (mm) with corresponding MIC values (mg/L) for rifabutin disk diffusion testing on CHE-MHA. Each *A. baumannii* isolate (n = 30) was tested using rifabutin disks at 5-, 10-, 20-, and 50-µg manually prepared from two drug products: RBT_1 (Thermo Scientific, Cat. No. 461210050, Lot A0451198) and RBT_2 (Thermo Scientific, Cat. No. 461210010, Lot A0451198). Rifabutin MICs were determined by agar dilution on PIH-supplemented MHA. Bar colors denote MIC categories, with yellow representing tentative WT isolates (MIC ≤0.0625 mg/L) and darker colors representing tentative NWT isolates with progressively higher MICs. No statistically significant difference in ZOI distributions was observed between rifabutin products, as determined by the Mann–Whitney U test (p >0.05).

## **Figure S3. ZOI–MIC correlations by CHE-MHA lot.**

Histograms show ZOI–MIC distributions for rifabutin disk diffusion on two lots of CHE-MHA prepared from Liofilchem® cations-depleted Mueller Hinton Broth (Cat. No. 400330; Lot Nos. 032524097 [Lot_1] and 041824033 [Lot_2]). Each *A. baumannii* isolate (n = 30) was tested using rifabutin disks at 5-, 10-, 20-, and 50-µg prepared manually from two drug products. Rifabutin MICs were determined by agar dilution on PIH-supplemented MHA. Bar colors denote MIC categories, with yellow representing tentative WT isolates (MIC ≤0.0625 mg/L) and darker colors representing tentative NWT isolates with progressively higher MICs. No statistically significant difference in ZOI distributions was observed between media lots, as determined by the Mann–Whitney U test (p >0.05).

## **Figure S4. ZOI–MIC correlations by rifabutin product source on MHA.**

Histograms show ZOI diameters (mm) with corresponding MIC values (mg/L) for rifabutin disk diffusion testing on standard MHA. Each *A. baumannii* isolate (n = 30) was tested using rifabutin disks at 5-, 10-, 20-, and 50-µg prepared manually from two drug products: RBT_1 (Thermo Scientific, Cat. No. 461210050, Lot A0451198) and RBT_2 (Thermo Scientific, Cat. No. 461210010, Lot A0451198). Rifabutin MICs were determined by agar dilution on PIH-supplemented MHA. Bar colors denote MIC categories, with yellow representing tentative WT isolates (MIC ≤0.0625 mg/L) and darker colors representing tentative NWT isolates with progressively higher MICs. No statistically significant difference in ZOI distributions was observed between rifabutin products, as determined by the Mann–Whitney U test (p >0.05).

## **Figure S5. ZOI–MIC correlations by MHA manufacturer.**

Histograms show rifabutin ZOI–MIC distributions for disk diffusion testing on standard MHA from two manufacturers: BD BBL™ (Cat. No. B12322) and Criterion (Cat. No. C7521). Each *A. baumannii* isolate (n = 30) was tested using rifabutin disks at 5-, 10-, 20-, and 50-µg prepared manually from two drug products. Rifabutin MICs were determined by agar dilution on PIH-supplemented MHA. Bar colors denote MIC categories, with yellow representing tentative WT isolates (MIC ≤0.0625 mg/L) and darker colors representing tentative NWT isolates with progressively higher MICs. No statistically significant difference in ZOI distributions was observed between manufacturers, as determined by the Mann–Whitney U test (p >0.05).

## **Figure S6. ZOI–MIC correlations by MHA lot (BD).**

Histograms show ZOI–MIC distributions for rifabutin disk diffusion on two lots of MHA from BD BBL™ (Cat. No. B12322; Lot Nos. 2343309 (Lot_1) and 4102223 (Lot_2)). Each *A. baumannii* isolate (n = 30) was tested using rifabutin disks at 5-, 10-, 20-, and 50-µg prepared manually from two drug products. Rifabutin MICs were determined by agar dilution on PIH-supplemented MHA. Bar colors denote MIC categories, with yellow representing tentative WT isolates (MIC ≤0.0625 mg/L) and darker colors representing tentative NWT isolates with progressively higher MICs. No statistically significant difference in ZOI distributions was observed between media lots, as determined by the Mann–Whitney U test (p >0.05).

## **Figure S7. ZOI–MIC correlations by MHA lot (Criterion).**

Histograms show rifabutin ZOI–MIC correlations for disk diffusion testing on two lots of MHA from Criterion (Cat. No. C7521; Lot Nos. 632186 [Lot_1] and 637817 [Lot_2]). Each *A. baumannii* isolate (n = 30) was tested using rifabutin disks at 5-, 10-, 20-, and 50-µg prepared manually from two drug products. Rifabutin MICs were determined by agar dilution on PIH-supplemented MHA. Bar colors denote MIC categories, with yellow representing tentative WT isolates (MIC ≤0.0625 mg/L) and darker colors representing tentative NWT isolates with progressively higher MICs. No statistically significant difference in ZOI distributions was observed between media lots, as determined by the Mann–Whitney U test (p >0.05).

# **References**

1.	Hindler, J. A. *et al.* Procedure for Confirming the Acceptability of Mueller-Hinton Agar Sources for Subsequent Use in CLSI and/or EUCAST Studies to Establish Disk Diffusion Quality Control Ranges. (2023).

2.	CLSI. M23 Ed6SEd2E | Procedure for Optimizing Disk Contents (Potencies) for Disk Diffusion Testing of Antimicrobial Agents Using Harmonized CLSI and EUCAST Criteria, 2nd Edition. *Clinical & Laboratory Standards Institute* https://clsi.org/standards/products/microbiology/documents/m23s/ (2020).

3.	Clinical and Laboratory Standards Institute (CLSI). Performance Standards for Antimicrobial Susceptibility Testing. 34th ed. CLSI supplement M100. (2024).
